# Functional investigation of the two ClpPs and three ClpXs in *Myxococcus xanthus* DK1622

**DOI:** 10.1128/msphere.00363-24

**Published:** 2024-08-27

**Authors:** Tianyu Wan, Ying Cao, Ya-jun Lai, Zhuo Pan, Yue-zhong Li, Li Zhuo

**Affiliations:** 1 State Key Laboratory of Microbial Technology, Institute of Microbial Technology, Shandong University, Qingdao, China; 2 Shenzhen Research Institute, Shandong University, Shenzhen, China; 3 Suzhou Research Institute, Shandong University, Suzhou, China; The University of Iowa, Iowa City, Iowa, USA

**Keywords:** ClpX, ClpP, protease, *Myxococcus xanthus*, social behavior, functional divergence

## Abstract

ClpXP is a protease complex that plays important roles in protein quality control and cell cycle regulation, but the functions of multiple ClpXs and multiple ClpPs in *M. xanthus* remain unknown. The genome of *Myxococcus xanthus* DK1622 contains two *clpP*s and three *clpX*s. The *clpP1* and *clpX1* genes are cotranscribed and are both essential, while the other copies are isolated in the genome and are deletable. The deletion of *clpX2* caused the mutant to be deficient in fruiting body development, while the *clpX3* gene is involved in resistance to thermal stress. Both ClpPs possess catalytic active sites, but only ClpP1 shows *in vitro* peptidase activity on the typical substrate Suc-LY-AMC. All of these *clpP* and *clpX* genes exhibit strong transcriptional upregulation in the stationary phase, and the transcription of the three *clpX* genes appears to be coordinated. Our results demonstrated that multiple ClpPs and multiple ClpXs are functionally divergent and may assist in the environmental adaptation and functional diversification of *M. xanthus*.

**IMPORTANCE:**

ClpXP is an important protease complex of bacteria and is involved in various physiological processes. *Myxococcus xanthus* DK1622 possesses two ClpPs and three ClpXs with unclear functions. We investigated the functions of these genes and demonstrated the essential roles of *clpP1* and *clpX1*. Only ClpP1 has *in vitro* peptidase activity on Suc-LY-AMC, and the isolated *clpX* copies participate in distinct cellular processes. All of these genes exhibited significant transcriptional upregulation in the stationary phase. Divergent functions appear in multiple ClpPs and multiple ClpXs in *M. xanthus* DK1622.

## INTRODUCTION

ClpXP is an AAA+ (ATPases Associated with diverse cellular Activities) protease complex that utilizes energy from ATP hydrolysis to control protein quality in numerous biological reactions and processes (1, 2). ClpXP consists of two distinct proteins, an AAA+ ATPase called ClpX and a peptidase with a Ser-His-Asp catalytic triplet called ClpP (3, 4). For the degradation function of the ClpXP complex, the hexamer ClpX recognizes substrates, then unfolds and transports them to the barrel-like tetradecamer degradation chamber, which consists of two axially stacked heptamer ClpP rings, for further hydrolysis (5–8). The substrates of ClpX differ in function; thus, the ClpXP protease is involved in various cellular physiological processes and plays an important role in the protein quality control of bacterial cells (9).

The protein sequences of ClpP and ClpX are relatively conserved in bacteria (5, 10). The *clpP* gene is often singly copied, as in *Escherichia coli* or *Bacillus subtilis*, but some bacteria, such as *Mycobacterium tuberculosis*, *Chlamydia trachomatis*, *Listeria monocytogenes*, *Clostridioides difficile*, *Pseudomonas aeruginosa,* and *Leptospira interrogans,* possess multiple *clpP* genes (10–14). In these bacteria, ClpP proteins are in different polymer forms, which are associated with their activities. Generally, the heptamer ClpP is inactive, while the homo- or heterotetradecamer shows peptidase activity. However, in *M. tuberculosis*, the homotetradecamers of the two ClpPs show no activity, and only the heterotetradecamer of ClpP1P2 is active (11). In contrast, ClpP1 and ClpP2 are active in the homotetradecamer form but not in the heptamer or heterotetradecamer form in *C. difficile* (13). Comparatively, multiple ClpXs are rare in bacteria (15).

*Myxococcus xanthus* is a gram-negative bacterium characterized by their unique social behavior (16, 17). Adapting to the complex life cycles, the bacterium has a large genome containing numerous duplicate genes, such as molecular chaperones. Previous work on the model strain *M. xanthus* DK1622 demonstrated that duplicate GroELs are functionally divergent (18–22) and that multiple DnaKs play different roles in cellular functions (23, 24). Besides, the Clp family proteins, ClpB and ClpC, were involved in the sporulation process and stress response in *M. xanthus* (25, 26). There are two copies of *clpP* and three copies of *clpX* in *M. xanthus* DK1622. In this study, we determined that *clpP1* and *clpX1* are in an operon and are both essential for cell growth, while other *clpP* and *clpX* genes are deletable. The deletion of nonessential *clpX* genes inhibits development or thermotolerance of this strain. ClpP1, but not ClpP2, exhibits peptidase activity on the typical substrate Suc-LY-AMC *in vitro*. We found that the *clpP* and *clpX* genes are transcriptionally upregulated at the stationary phase, and the deletion of the isolated *clpX2* gene causes the upregulation of other *clpX* copies. Our results show the functional divergence of two ClpPs and three ClpXs in *M. xanthus*.

## RESULTS

### Bioinformatics analysis of the two ClpPs and three ClpXs in *M. xanthus* DK1622

**Fig 1:**
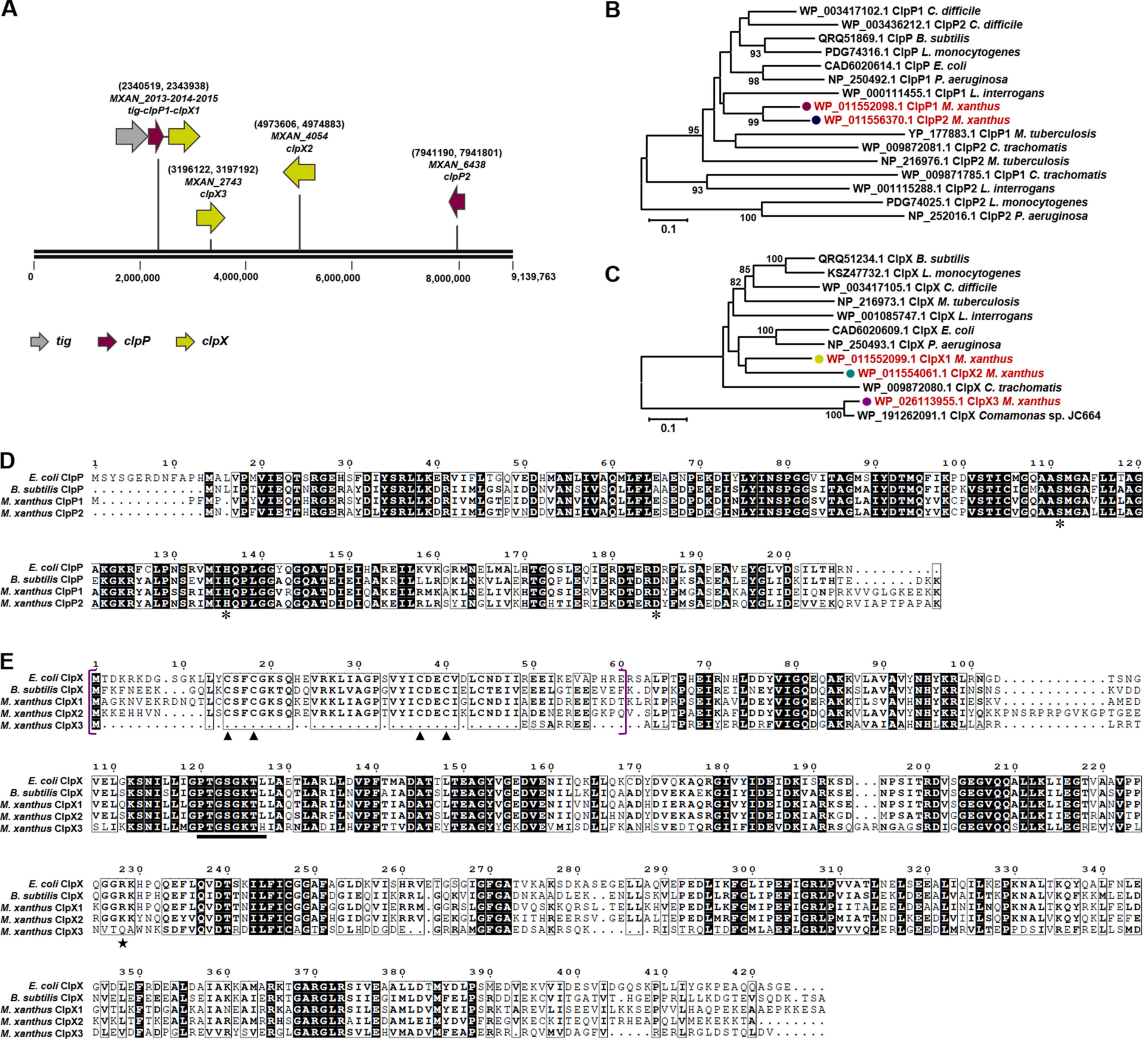
Bioinformatic analysis of multiple ClpPs and ClpXs in *M. xanthus* DK1622. (**A**) The locations of *clpP1* (*MXAN_2014*), *clpP2* (*MXAN_6438*), *clpX1* (*MXAN_2015*), *clpX2* (*MXAN_4054*), and *clpX3* (*MXAN_2743*) in the genome of *M. xanthus* DK1622. Phylogenetic tree of ClpP (**B**) and ClpX (**C**) proteins in *E. coli*, *B. subtilis*, *M. tuberculosis*, *P. aeruginosa*, *C. difficile*, *M. tuberculosis*, *L. interrogans*, *C. trachomatis*, *L. monocytogenes*, and *M. xanthus* DK1622. (**D**) Sequence alignment of ClpP proteins of *E. coli*, *B. subtilis*, and *M. xanthus* DK1622. Furthermore, 100% conserved amino acid residues in these proteins are highlighted with a black background, less conserved residues are in the frame, and residues with similar properties are presented in bold font. The conserved amino acids of the catalytic triad are marked by *. (**E**) Sequence alignment of ClpX proteins of *E. coli*, *B. subtilis*, and *M. xanthus* DK1622. The N-terminal domain regions are shown in purple brackets. The “▲“ symbol marks the Cys residues required for dimerization. The “★” symbol indicates the Arg residues in the GFR motif. The black bar marks the ATP-binding site.

According to the NCBI genome annotation, *M. xanthus* DK1622 has two *clpP* and three *clpX* copies. The two ClpP proteins are relatively conserved, with 79.59% identity to each other and 65.97% (ClpP1, MXAN_2014) and 65.24% (ClpP2, MXAN_6438) identity to *E. coli* ClpP (EcClpP). Similarly, the identities of the three ClpX proteins ranged from 41.98% to 64.79% and were 67.24% (ClpX1, MXAN_2015), 64.82% (ClpX2, MXAN_4054), and 43.54% (ClpX3, MXAN_2743) identical to those of *E. coli* ClpX (EcClpX) (Table S1). There also exist eight copies of *clpA*/*B*/*C* in *M. xanthus* DK1622, whereas their proteins are less conserved (Table S1). The genes encoding ClpP1 and ClpX1 appear to form an operon with the *tig* gene (*MXAN_2013*, the coding gene of the ribosome-associated molecular chaperone trigger factor) in the order *tig-clpP-clpX*, whereas the other *clpP* and *clpX* copies are isolated in the genome (Fig. 1A). Phylogenetic analysis of the ClpP and ClpX proteins with those in other bacteria revealed that *M. xanthus* ClpP1 and ClpP2 are close to each other and are clearly separated from other ClpP proteins (Fig. 1B). *M. xanthus* ClpX1 and ClpX2 are highly similar in sequence, while ClpX3 is less conserved to ClpX1/X2 (Fig. 1C). These results suggested that ClpP2 is likely duplicated from ClpP1, while ClpX3 is probably a horizontally transferred gene from other bacteria.

Protein sequence alignment revealed that, similar to *B. subtilis* ClpP but not *E. coli* ClpP, the *M. xanthus* ClpP1 and ClpP2 proteins both have no leader sequence at the N-terminus (Fig. 1D), which is rapidly cleaved *in vivo* to yield the native enzyme in *E. coli* (27). *M. xanthus* ClpP1 and ClpP2 both have classic Ser-His-Asp catalytic triads. Comparatively, ClpX1 and ClpX2 have the characteristic N-terminal domain with Cys residues, which is required for dimerization (28, 29), while ClpX3 lacks the N-terminal domain (Fig. 1E).

### Neighboring *clpP1* and *clpX1* are both essential genes in *M. xanthus*

**Fig 2:**
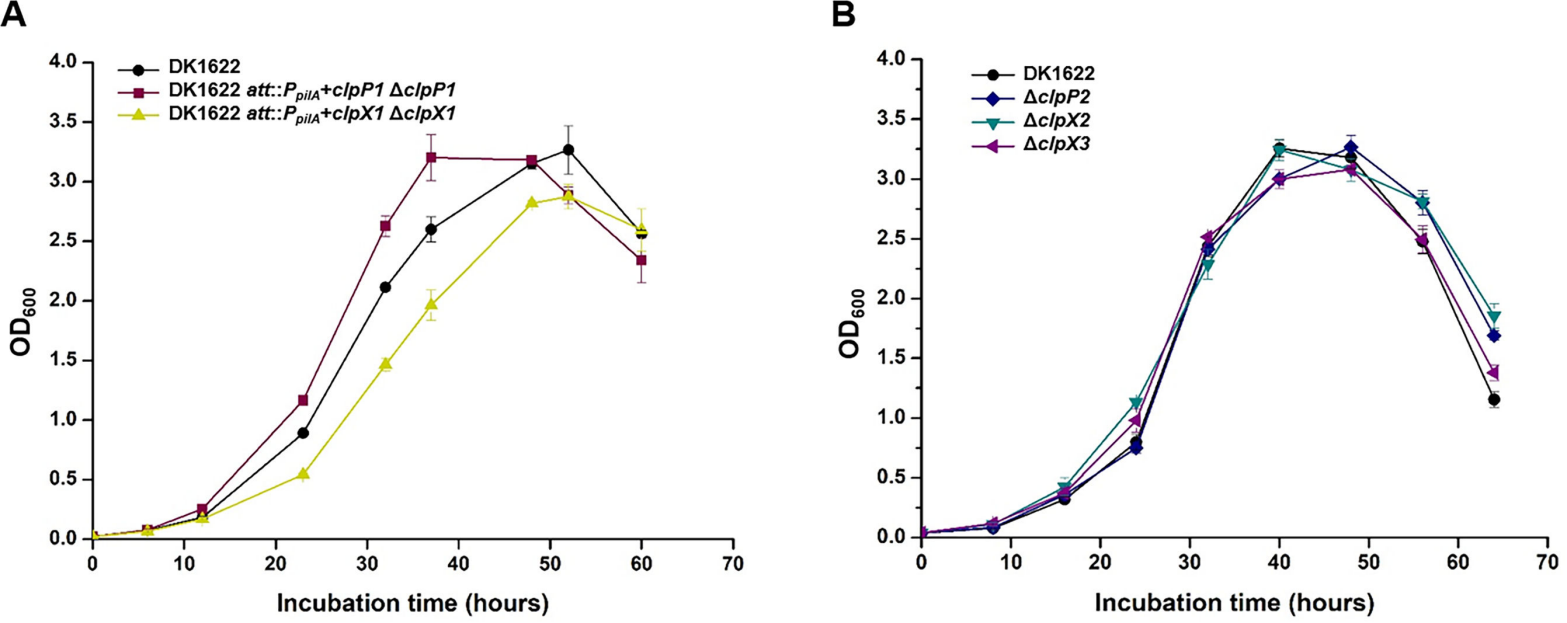
Growth of single-deletion mutants of multiple *clpP*s and *clpX*s in *M. xanthus* DK1622. (**A**) Growth curve of *clpP1* or *clpX1* deletion mutants based on the corresponding *attB* site recombination strain. (**B**) Growth curves of *clpP2*, *clpX2*, and *clpX3* deletion mutants under normal conditions.

To explore the cellular functions of multiple ClpPs and ClpXs in *M. xanthus* DK1622, we attempted to knock out each gene but were unable to delete *clpP1* or *clpX1*. To confirm the essentiality of these two genes, we used the site-specific recombination plasmid pSWU30 to complement *clpP1* or *clpX1* at the *attB* site of the genome. Subsequently, we attempted to delete the original *clpP1* or *clpX1* from the corresponding complemented strain and, as expected, obtained viable mutants. The growth curves of these two mutants were similar to that of the wild-type strain DK1622 (Fig. 2A). Thus, the neighboring *clpP1* and *clpX1* genes are essential for the survival of *M. xanthus* DK1622 cells. Notably, RT-PCR verified that *tig*, *clpP1,* and *clpX1* are cotranscribed and form an operon (Fig. S1); however, the *tig* gene located in the operon *tig-clpP-clpX* could be deleted. Further pulldown results indicated that these three proteins can interact with each other in pairs (Fig. S2), suggesting a coordinated relationship between transcription and function among the proteins encoded by the *tig-clpP1-clpX1* operon. In contrast, the *clpP2*, *clpX2,* and *clpX3* genes are isolated in the genome and are deletable. The knockout mutant of *clpP2*, *clpX2,* or *clpX3* also had nearly the same growth curve as the wild-type strain DK1622 under normal conditions (Fig. 2B).

### The isolated *clpX* copies are involved in specific physiological processes

**Fig 3:**
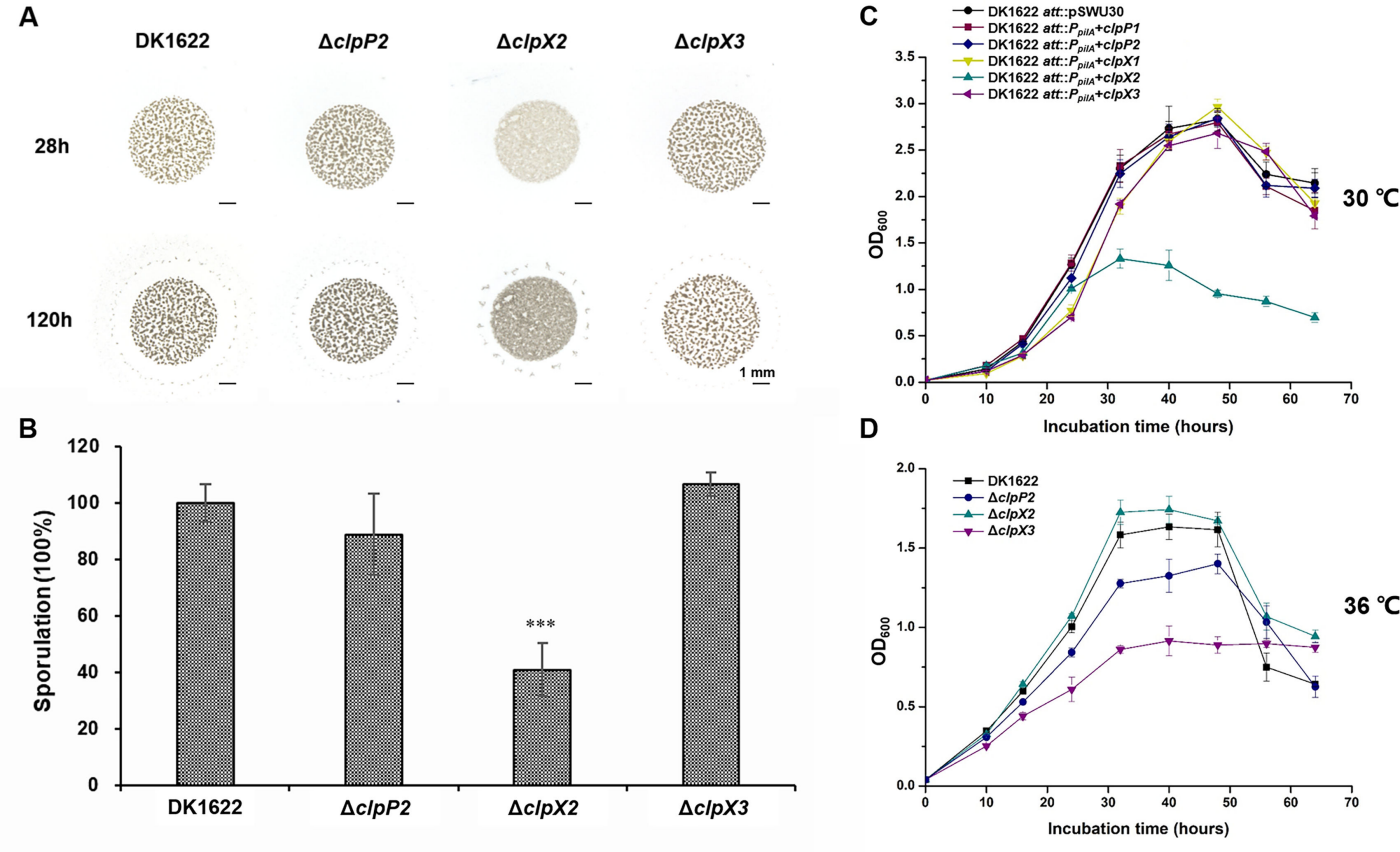
Cellular physiological function analysis of *clpP2*, *clpX2*, or *clpX3* deletion mutants. (**A**) Fruiting body formation of these deletion mutants. The black bar is 1 mm. (**B**) The sporulation ability of the deletion mutants compared to that of the wild-type strain DK1622. For statistical analysis, ***, *t* test, *P* value <0.001. (**C**) Growth of *M. xanthus* DK1622 with the overexpression of these *clpP*s and *clpX*s under normal conditions. (**D**) Growth curves of *clpP2*, *clpX2*, and *clpX3* deletion mutants under thermal stress conditions (36°C).

*M. xanthus* cells have two motility systems: system A is required for the movement of single cells, and system S is mainly involved in the movement of cells in groups (17). We assayed the motilities of the knockout mutants of *clpP2*, *clpX2,* and *clpX3* using DK1622, the Δ*aglZ* (A^−^ S^+^) mutant, and the Δ*pilA* (A^+^ S^−^) mutant as controls. Single and grouped cells of these mutants both moved like wild-type cells on 1.5% agar (Fig. S3) and 0.4% agar plates (Fig. S4), respectively, indicating that this *clpP* or *clpX* did not affect the motilities of *M. xanthus*. Similarly, the predation abilities of these mutants on *E. coli* mats did not differ from those of the wild-type strain (Fig. S5). However, different phenomena were found in the development of the knockout mutants on the non-nutritive TPM plate. The aggregation and spore formation of the Δ*clpP2* and Δ*clpX3* strains were similar to those of the wild-type strain, but the cells of the *clpX2* deletion strain did not completely form the fruiting body structure (Fig. 3A). The sporulation rate of the Δ*clpX2* mutant was significantly decreased, approximately 41.0% of that of the wild-type strain (*t* test, *P* value <0.001; Fig. 3B).

We also used the site-specific recombinant plasmid pSWU30 and the *pilA* promoter [P*_pilA_*, strong promoter used in *M. xanthus* (22, 30)] to construct the overexpression mutants of each of the two *clpP* and three *clpX* genes. These mutants had a normal growth curve under normal condition (30°C) as the wild-type strain with the empty pSWU30 plasmid, except the *clpX2*-overexpressing mutant, which was inhibited (Fig. 3C). The ClpXP protease plays an important role in the degradation of denatured proteins (31). We assessed the growth of these knockout mutants at a high temperature (36°C) and found that the growth of the Δ*clpX3* mutant was significantly weaker than that of the *clpP2* or *clpX2* knockout mutant as well as the wild-type strain DK1622 (Fig. 3D). The above results suggested that although the deletion of *clpP1*, *clpX1,* or *clpX2* had no effect on motility or predation behavior, *clpX2* was involved in the characteristic sporulation and fruiting body formation of myxobacteria, while *clpX3* was involved in resistance to thermal environmental stress.

**Fig 4:**
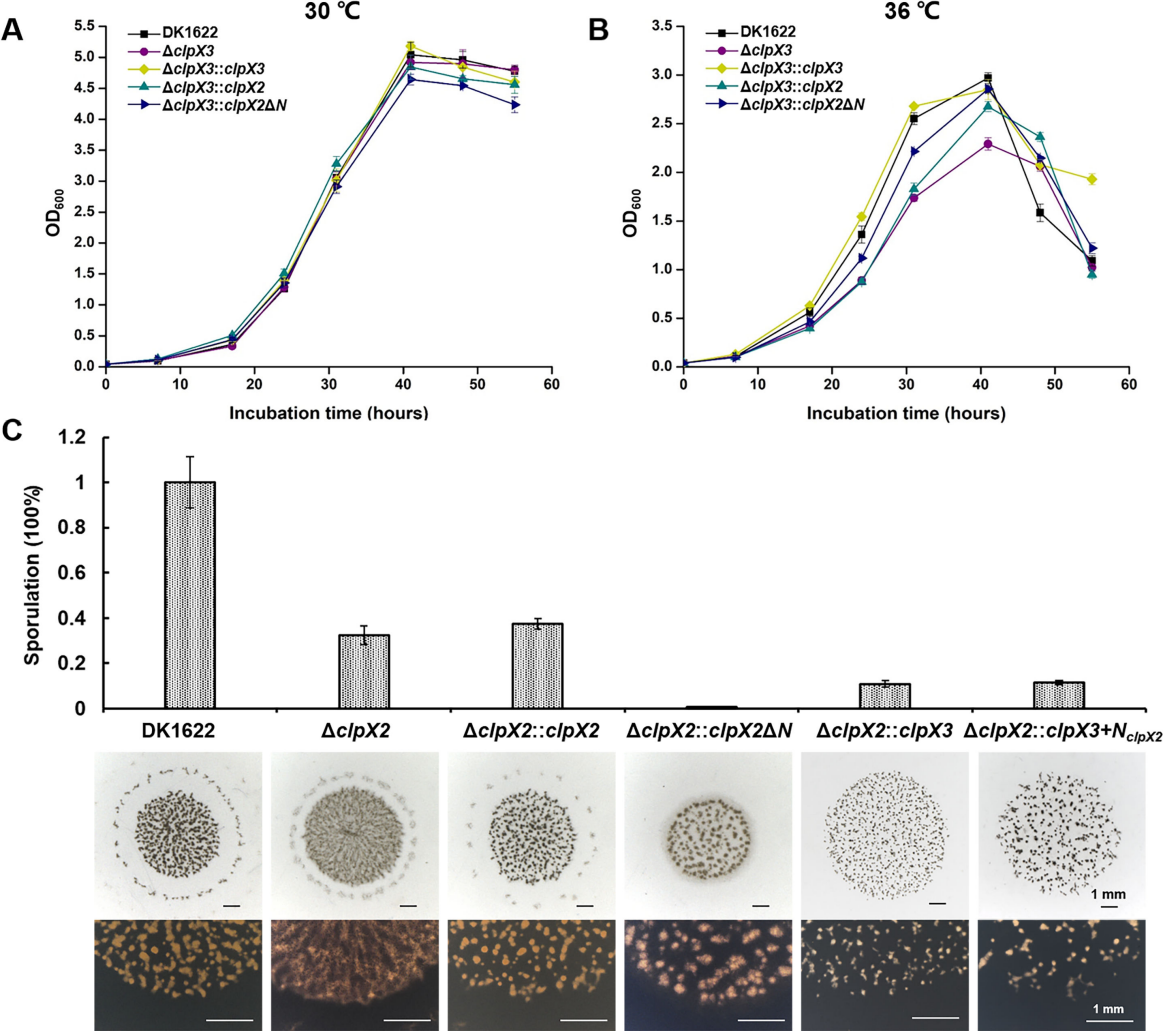
Phenotypic analysis of complementation strains of ClpX2, ClpX3, and their variants. Growth curves of complementation strains based on *clpX3* deletion mutant under normal conditions (**A**) and thermal stress conditions (**B**). (**C**) Sporulation and fruiting body formation of complementation strains based on *clpX2* deletion mutant. The black and white bars are 1 mm.

We further complemented the corresponding gene with the P*_pilA_* promoter in the *clpX2* or *clpX3* deletion mutant. The complemented genes were integrated at the *attB* site of the genome by using pSWU19. The *clpX3* complementation strain (Δ*clpX3::clpX3*) rescued the growth defect at 36°C compared to the *clpX3* deletion mutant (Fig. 4A and B). However, the compensation of ClpX2 (Δ*clpX2::clpX2*) restored the fruiting body formation defect of the *clpX2* deletion mutant, but sporulation was still defective (Fig. 4C), possibly due to inadequate transcriptional compensation between the P*_pilA_*-driven *clpX2* copy and the original *clpX2* gene during sporulation (32). Furthermore, we constructed complemented strains of *clpX2* (Δ*clpX3::clpX2*) or the N-terminal truncated *clpX2* (Δ*clpX3::clpX2*Δ*N*) in the *clpX3* deletion mutant, with ClpX2 possessing a complete N-terminal domain while ClpX3 lacking the domain (Fig. 1E). The complementation of ClpX2 and ClpX2ΔN both restored the growth at 36°C of *clpX3* deletion mutant to some extent (Fig. 4A and B). Notably, the N-terminal deleted variant, ClpX2ΔN, showed a more pronounced effect. Interestingly, compensating for the *clpX2* deletion mutant with ClpX2ΔN (Δ*clpX2::clpX2*Δ*N*) resulted in a more serious sporulation rate defect (Fig. 4C). On the other hand, although the complementation of *clpX3* (Δ*clpX2::clpX3*) or its chimera with N-terminal domain of ClpX2 (Δ*clpX2::clpX3 + N _clpX2_*) partially rescued fruiting body formation, they both failed to restore the sporulation rate of the *clpX2* deletion mutant (Fig. 4C). These results suggested that there exists some functional compensation between ClpX2 and ClpX3, and their cellular functions are probably associated with their N terminals.

### *M. xanthus* ClpP1, but not ClpP2, has peptidase activity *in vitro*

**Fig 5:**
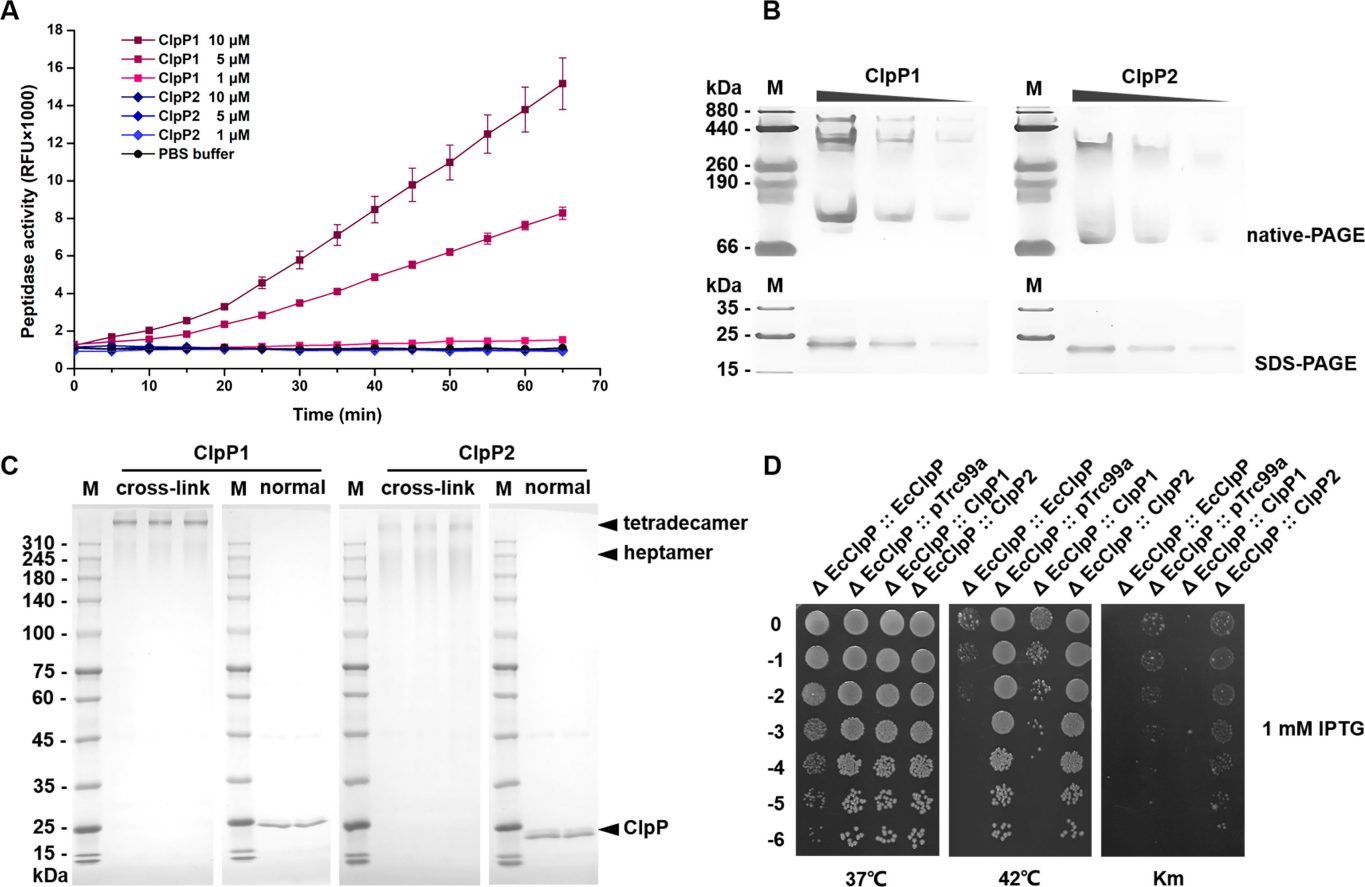
Differences in the activity and polymer form of the two *M. xanthus* ClpP proteins. (**A**) The peptide cleavage activity of ClpP1 and ClpP2 on Suc-LY-AMC. (**B**) Native-PAGE of nonlabeled ClpP1 or ClpP2 on 8% gel and SDS-PAGE on 10% gel. (**C**) SDS-PAGE of cross-linked ClpP1 or ClpP2 by 1% glutaraldehyde. (**D**) Growth of *E. coli* recombinant mutants heterologously expressing ClpP1 and ClpP2 under different conditions. Also, 1:10 serial dilutions (vertical direction) were spotted on Luria-Bertani plates containing 100 µg/mL Amp with 1 mM IPTG.

As shown in Fig. 5A, with the increase of ClpP1 protein concentration in the reaction system, the cleavage rate of Suc-LY-AMC clearly increased. However, there was no cleavage of Suc-LY-AMC by ClpP2, even when the protein concentration increased to 10 µM. The *in vitro* activity of the ClpP protease is related to its tetradecamer or heptamer form (10, 33, 34). Native PAGE of the purified ClpP proteins showed that ClpP1, but not ClpP2, had a large stable polymer form (Fig. 5B). Further protein cross-linking experiments demonstrated that the tetradecamer band was the dominant form of ClpP1 *in vitro*, while the corresponding band of ClpP2 was weaker (Fig. 5C). Native-PAGE and cross-linking results suggested that the polymeric difference of the ClpP1 and ClpP2 proteins may be the main reason for the presence of the active or inactive form of the ClpP protease.

To further determine the functional conservation of the two *M. xanthus* ClpPs, we knocked out the original *clpP* gene from *E. coli* MG1655 and complemented *M. xanthus clpP1* or *clpP2* in the pTrc99a plasmid containing an isopropyl-beta-D-thiogalactopyranoside (IPTG)-inducible promoter (Fig. S6). EcClpP- and empty pTrc99a-complemented strains were used as references. After the induction by 1 mM IPTG, all these complemented strains grew normally at 37°C. However, under thermal condition (42°C), the growth of the ClpP1-overinduced strain was severely inhibited, which was similar to that of the EcClpP-overinduced strain, while the growth of the ClpP2-overinduced strain was not inhibited, which was similar to that of the empty pTrc99a-complemented strain (Fig. 5D). Although ClpP is a heat-shock protein, the overinduced ClpP protein in high temperature might potentially cause the dysregulation of intracellular protein degradation. A similar differential inhibition phenomenon occurred in the presence of 3 µg/mL kanamycin. These results indicated that *M. xanthus* ClpP1, but not ClpP2, had a function similar to that of EcClpP.

We also deleted the original *clpX* gene from *E. coli* MG1655 and complemented it with each *M. xanthus clpX* gene via the pTrc99a plasmid. None of the overexpression mutants exhibited significant differences in growth under different conditions (Fig. S7).

### Multiple *clpP* and *clpX* genes all upregulated in the stationary phase

**Fig 6:**
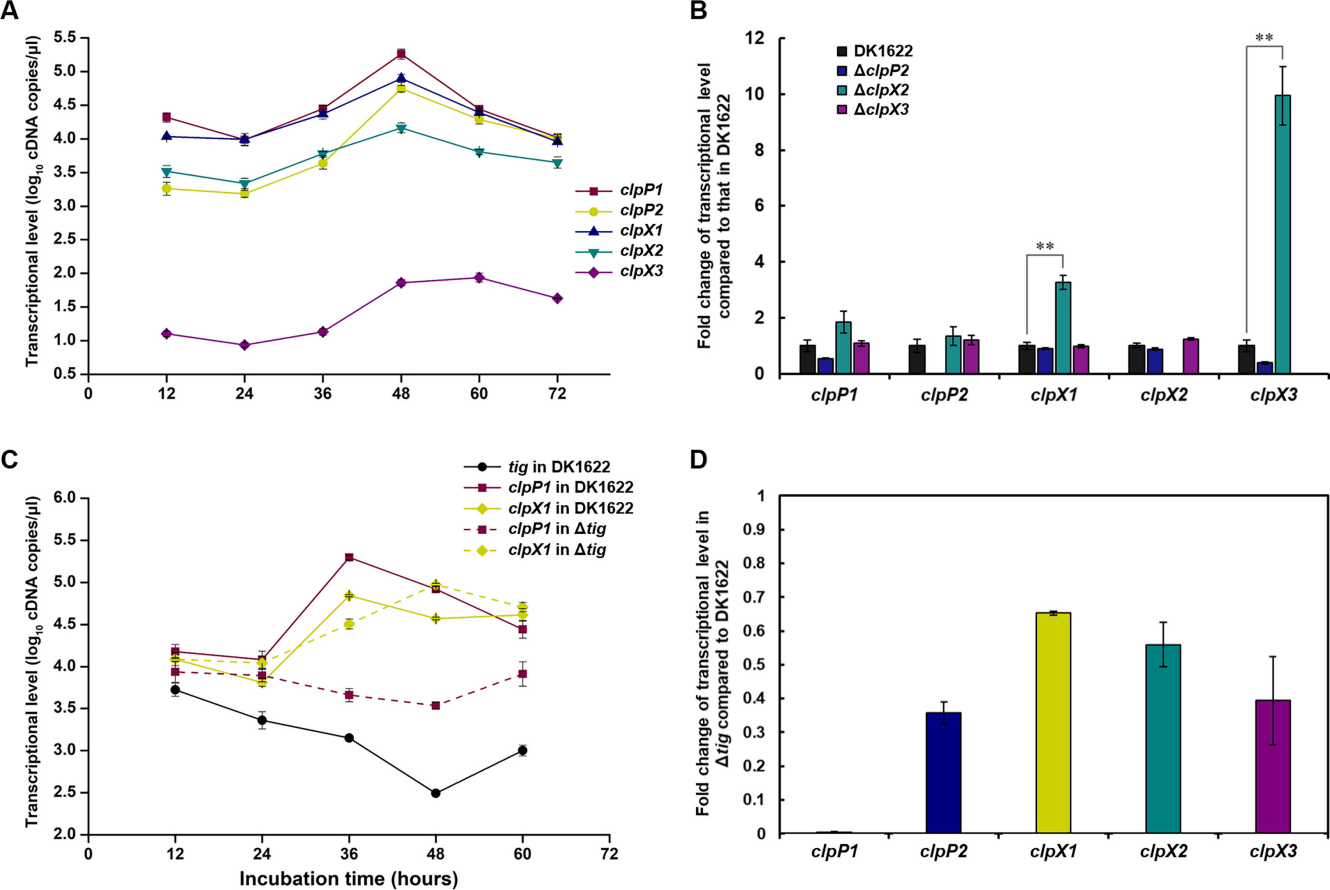
Transcriptional analysis of multiple *clpP*s and *clpX*s in *M. xanthus* DK1622. (**A**) Changes in the transcription of these *clpP* and *clpX* genes in the wild-type strain during incubation. (**B**) Transcriptional fold changes of these genes in different deletion mutants at 42 h compared to those in DK1622. The transcriptional levels of each gene in DK1622 were set as 1. For statistical analysis, **, *t* test, *P* value <0.01. (**C**) Changes in the transcription of *clpP1* and *clpX1* in *M. xanthus* DK1622 and the *tig* deletion mutant at different time points. (**D**) Transcription fold changes of these genes in the *tig*-deleted mutant compared to those in DK1622 at 42 h. The transcriptional levels of each gene in DK1622 were set as 1.

We analyzed the transcription of these *clpP* and *clpX* genes. The essential *clpP1* and *clpX1* had the highest transcription, *clpP2* and *clpX2* had similar but slightly lower transcription, and *clpX3* had extremely low transcription (Fig. 6A). According to the growth curve of *M. xanthus* DK1622 (Fig. 2B), the 12- and 24-h liquid incubation time points were in the logarithmic growth period, 36 and 48 h were in the stationary phase, and finally, the time points at 60 and 72 h indicated cell lysis because the OD values dropped. The five *clpP* and *clpX* genes had similar transcriptional trends during incubation and were significantly upregulated at 36–48 h. The upregulated fold changes for *clpP1*, *clpP2*, *clpX1*, *clpX2*, and *clpX3* were as follows: 6.47-fold, 12.83-fold, 3.38-fold, 2.41-fold, and 5.37-fold, respectively. The upregulation of these *clpP* and *clpX* genes in *M. xanthus* at the stationary phase is consistent with the finding that ClpXP is an important protease for maintaining proteome homeostasis in the stationary phase (35, 36).

We also measured the transcriptional levels of these genes in knockout mutants at 42 h (the stationary phase). The transcriptional level of each of the *clpX* and *clpP* genes in the wild-type strain DK1622 was set to 1, and the fold change of their transcriptional levels in different knockout mutants was calculated. The results showed that the transcription of the essential *clpX1* gene was upregulated 3.3-fold (*t* test, *P* value <0.01), and the least conserved *clpX3* gene was upregulated 10.0-fold (*t* test, *P* value <0.01) in the Δ*clpX2* mutant (Fig. 6B). Combined with the phenotypic analysis of ClpX2 and ClpX3 complementation strains, the transcriptional correlation suggested again that potential functional compensation existed among the multiple *clpX* copies.

For the cotranscribed *tig-clpP1-clpX1*, we found that the transcription of *clpP1* in Δ*tig* was significantly downregulated in the stationary phase compared to that in DK1622, but the transcription of *clpX1* did not change obviously (Fig. 6C). At the 42-h time point of incubation, compared to that of DK1622, the transcription of *clpP1* was significantly downregulated to 0.5% in Δ*tig*, and *clpP2* was also downregulated by 2.8-fold (Fig. 6D). This result suggested that the deletion of *tig* resulted in a significant transcriptional decline of the adjacent *clpP1*gene, but not the neighbor *clpX1* gene, and also led to the downregulation of the distantly located *clpP2* gene.

## DISCUSSION

The occurrence of gene duplication can provide genetic materials for new functional genes to organisms (37). The evolutionary fates of duplicate genes include pseudogenization, conservation of gene function, subfunctionalization, and neofunctionalization; ultimately, the retained duplicate genes are always beneficial for organisms (38, 39). Myxobacteria have a large number of multiplied genes that are involved in their complex life cycle (40), adapting to diverse environments (41). We have previously reported the functional divergence of multiple GroELs and DnaKs and the different preferences for social behavior in *M. xanthus* (18, 23). Here, we investigated the functions of two ClpP and three ClpX copies in *M. xanthus* DK1622. ClpP1 and ClpX1 of the *tig-clpP-clpX* operon are both essential in *M. xanthus*. Although the transcription of *clpP1* decreased approximately 200-fold in the Δ*tig* mutant, the growth of the strain was not affected, indicating that the dependence on *clpP1* for cells is probably not caused by its high transcription. Interestingly, although ClpP2 did not show peptidase activity *in vitro* and its deletion did not affect growth, its encoded gene still had a high transcriptional level, with transcription upregulated 36.3-fold in the stationary phase. In addition to *M. xanthus*, inactive ClpP was also found in *C. trachomatis*, *L. monocytogenes,* and *P. aeruginosa* with two copies of ClpPs (10, 33, 34). In *L. monocytogenes*, ClpP1 alone only forms an inactive heptamer, but the heterotetradecamer of ClpP1P2 is active. In *Streptomyces*, one of the two conserved ClpP copies can bind to ClpX to open axial pores for substrate degradation, while the other ClpP has no binding capacity to ClpX but binds to the intracellular antibiotic acyldepsipeptides for activation (42). Thus, *M. xanthus* ClpP2, which lacks classical peptidase activity *in vitro*, may cooperate with ClpP1 to form a heterotetradecamer or be activated by certain compounds to perform protease functions.

The three copies of ClpX in *M. xanthus* DK1622 differ in sequence, and ClpX3 lacks the characteristic N-terminal domain, the region for dimerization (28, 29). A mutation in the key residue R of the RKH motif, associated with substrate recognition of the ClpX protein, affects the recognition of SsrA-labeled substrates (43, 44). This key substrate recognition residue R is conserved in *E. coli* ClpX, *B. subtilis* ClpX, and *M. xanthus* ClpX1, but it is replaced by K in ClpX2, and ClpX3 has no RKH motif. Unfortunately, the ClpX2 and ClpX3 proteins were aggregated into sediment in solution after the solubilization label was removed, so we could not obtain the ClpX2 and ClpX3 proteins for further *in vitro* detection.

The transcription of *clpX3* was extremely low among *M. xanthus clpX* copies. However, this gene was upregulated 8.4-fold at the later growth period compared to the logarithmic period and 10.0-fold in the Δ*clpX2* mutant compared to the wild-type strain. In addition, the growth of the Δ*clpX3* mutant was significantly inhibited at high temperature. These results suggested that although the protein sequence of ClpX3 is highly different from that of classic ClpX and that the *clpX3* gene is far less transcribed than other *clpX/P* genes are, ClpX3 still has certain cellular functions related to environmental adaptation. Another isolated copy of ClpX, ClpX2, appears to regulate the specific fruiting body formation of *M. xanthus*. The motility of the Δ*clpX2* mutant was consistent with that of the wild-type strain, indicating that the effect of *clpX2* on development was unrelated to motility. Additionally, the absence of *clpB* (*MXAN_5092*) or *clpC* (*MXAN_4832*) belonging to the Clp family in *M. xanthus* also has impact on the development and sporulation processes of myxobacteria (25, 26). In summary, these multiple ClpP and ClpX copies have evolved divergent functions to assist in adapting to the complex life cycle and environmental adaptation of *M. xanthus* cells.

## MATERIALS AND METHODS

### Bioinformatic analysis

The genome annotations of these two ClpP proteins, ClpP1 (MXAN_2014) and ClpP2 (MXAN_6438), and three ClpX proteins, ClpX1 (MXAN_2015), ClpX2 (MXAN_4054), and ClpX3 (MXAN_2743), in *M. xanthus* DK1622 were downloaded from NCBI. Sequence alignment was performed by using the online program MAFFT (https://www.ebi.ac.uk/Tools/msa/mafft/), and the sequences were analyzed with ESPript (https://espript.ibcp.fr/ESPript/cgi-bin/ESPript.cgi). The phylogenetic tree was constructed based on protein sequences by using MEGA 11.

### Strains and growth conditions

The strains and plasmids used are listed in Table S2. *M. xanthus* strains were cultured in nutrient-rich casitone-based (CTT) media [1% casitone, 8 mM MgSO_4_, 10 mM Tris-HCl (pH 7.6), 1 mM K_2_HPO_4_-KH_2_PO_4_; pH 7.6] (45) at 30°C, while *E. coli* strains were routinely cultured in Luria-Bertani (LB) media (1% tryptone, 0.5% yeast extract, 1% NaCl) at 37°C. Agar (1.5%) was added to the solid plates. For selection, ampicillin (Amp) was added to the medium at a final concentration of 100 µg/mL, kanamycin (Km) at a final concentration of 40 µg/mL, or tetracycline (Tet) at a final concentration of 10 µg/mL unless otherwise noted.

### Construction of plasmids and strains

The primers used for PCR amplification are listed in Table S3.

*E. coli* mutant strains were constructed using a previously described method. First, the original *clpP* or *clpX* gene was deleted according to a method described previously (46). Then, we cloned the *clpP1*, *clpP2*, *clpX1*, *clpX2*, *clpX3*, and the original *clpP* and *clpX* genes from *E. coli* into the plasmid pTrc99a using the ClonExpress II One Step Cloning Kit (Vazyme) to construct the recombinant expression plasmids. Afterward, the recombinant expression plasmids were transformed into *clpP-* or *clpX*-deleted *E. coli* MG1655 mutants to construct recombinant strains.

Gene deletion in *M. xanthus* DK1622 was performed by using positive-negative KG cassettes as previously described (47). The deletion plasmids were constructed by cloning 800 bp upstream and downstream homologous fragments of the gene into pBJ113. The deletion plasmid was transformed into DK1622 cells by electroporation, colonies with Km resistance were first selected, and then, 1.5% D-galactose (Sigma) was used for the second selection. Finally, the deletion mutants were confirmed by PCR and sequencing.

The *M. xanthus* DK1622 overexpression mutants were constructed by combining the *clpP* and *clpX* genes of *M. xanthus* DK1622 after the P*pilA* promoter and then cloning the combined sequences into the *attB* site-specific recombination plasmid pSWU30, which is usable in *Myxococcus*. The overexpression plasmids were also transformed into *M. xanthus* DK1622 cells by electrotransfer. A single colony with Tet resistance was selected and evaluated.

### Growth curves under different conditions

The *M. xanthus* strains were cultured in liquid CTT medium at 30°C and 200 rpm for approximately 20 h as seed cells, at which time the OD_600_ value was approximately 1 (logarithmic phase). The cells were then transferred to 50 mL of CTT liquid medium with a final concentration of OD_600_ = 0.04 and cultured at 30°C and 200 rpm, and a small part of the culture medium was removed every 8 h to measure the OD_600_. For measurement of the growth curve under pressure, 36°C was selected as the thermal pressure culture condition.

### Motility assays

This assay was performed according to a previous method (17). The *M. xanthus* strains were cultured to the logarithmic phase and centrifuged to collect the cells. The cells were washed three times with TPM buffer (10 mM Tris-HCl, 8 mM MgSO_4_, 1 mM K_2_HPO_4_- KH_2_PO_4_; pH 7.6) and adjusted to a final concentration of 5 × 10^9^ cells/mL (adjust to OD_600_ = 1 and then 35 times concentrated). Two-microliter droplets were cultured on 1.5% CTT solid plates and 0.3% CTT solid plates at 30°C.

### Predation assays

The predation assay was conducted as previously described (23). The concentrations of *M. xanthus* and *E. coli* were adjusted to 5 × 10^9^ and 1 × 10^11^ cells/mL (adjust to OD_600_ = 1 and then 100 times concentrated), respectively, by TPM. Thirty-five microliters of *E. coli* cells were dropped onto a TPM plate to form a colony approximately 1 cm in diameter. Then, 5 µL of *M. xanthus* was dropped into the center of the *E. coli* colony. When cultured at 30°C, the rate of *M. xanthus* expansion was used to characterize the predation ability of *M. xanthus* on *E. coli*.

### Fruiting body formation assays

The detection of fruiting body formation of *M. xanthus* was performed according to a previous study (18). Eight microliters of *M. xanthus* cells at a concentration of 5 × 10^9^ cells/mL were dropped onto a non-nutritive TPM plate and cultured at 30°C for 5 days. The fruiting bodies were observed by stereoscopic microscopy. Subsequently, the fruity bodies were dispersed in 1 mL of TPM and heated at 50°C for 2 h to kill the vegetative cells. After ultrasonic treatment, the spore suspension was diluted on CTT plates and cultured at 30°C. The rates of sporulation were calculated by the number of colonies growing on the CTT plates.

### Protein purification

The protein purification procedure was performed according to a previous method (48). The gene fragments were amplified by Phanta Super-Fidelity DNA Polymerase (Vazyme) and then inserted into the pET28a plasmid or pMal-c5x plasmid to construct protein expression vectors. These vectors were subsequently transformed into *E. coli* BL21 for protein expression. Recombinant *E. coli* strains were cultured in liquid LB supplemented with 100 µM IPTG to induce protein expression at 16°C for approximately 20 h. The cells were collected and ultrasonically crushed after being resuspended and then centrifuged at 12,000 rpm at 4°C for 30 min. The supernatants were collected. The proteins expressed by the recombinant pET28a plasmids were fused with a His-tag, so they were bound to Ni Sepharose High Performance, the unbound proteins were washed off with suspension buffer, and then, the proteins were eluted with suspension buffer supplemented with gradient concentrations of imidazole. The proteins expressed by the pMal-c5x recombinant plasmids were fused with the solubility-enhancing tag MBP, the unbound proteins were washed off with suspension buffer, and the proteins were then eluted with 10 mM maltose. The fusion proteins with MBP were digested by the enzyme to obtain pure proteins.

### Pulldown assay for protein-protein interaction

The pulldown assay of purified proteins with His or MBP tag was performed following a previously established method (49). The two proteins, each labeled with different tag, were mixed and incubated at room temperature for 30 min. Subsequently, the mixture was passed through the Ni-NTA resin, allowing the His-labeled proteins as well as the MBP-labeled proteins that could interact with His-labeled proteins to bind to the Ni-NTA resin. The unbound proteins were washed off using suspension buffer, while the bound proteins were eluted using wash buffer supplemented with 250 mM imidazole. The sample that MBP protein mixed with His-labeled protein was used as the negative control.

### Peptidase assays of *M. xanthus* ClpPs

The peptidase assays were performed according to previous methods (12). The commonly used fluorogenic dipeptide N-succinyl-Leu-Tyr-AMC (S1: Suc-LY-AMC; Sigma) was used as the substrate. The reaction was carried out in phosphate-buffered saline (50 mM phosphate buffer, 100 mM KCl; pH 7.6) at 25°C. Each reaction system contained 100 µM Suc-LY-AMC and different concentrations of nontagged ClpP1 or ClpP2. The hydrolysis of the fluorogenic dipeptide was measured in an Infinite M200Pro plate reader (Tecan) at an excitation wavelength of 380 and emission wavelength of 460 nm.

### Native-PAGE detection

The native-PAGE system was precooled on ice, and the experiment was carried out at 4°C to avoid protein denaturation. Tris-Glycine Native PAGE Running Buffer, pH 8.8, was purchased from Sangon. ClpP1 and ClpP2 proteins with no tag were incubated for 30 min on ice and separated by 10% PAGE under 10 mA electricity for 3 h.

### Cross-linking of *M. xanthus* ClpPs

The cross-linking of ClpP1 or ClpP2 was performed as previously described (50). ClpP1 and ClpP2 proteins were incubated at the same concentration of 20 µM in HEPES buffer (20 mM HEPES, 100 mM NaCl, and 1 mM EDTA; pH 7.5) for 30 min at 25°C. The reaction was initiated at 25°C by adding glutaraldehyde (Sigma) to a final concentration of 0.1% and quenched after 10 min with 200 mM Tris-HCl, pH 7.5, for 15 min. The completed reaction mixtures were detected by SDS-PAGE.

### Quantitative PCR analysis

Quantitative PCR analysis was carried out as described previously (23). Cellular RNA was obtained with a Bacterial Total RNA Extraction Kit (Thermo Fisher, USA.). Then, reverse transcription was carried out with HiScript Q-RT SuperMix for qPCR (Vazyme) after genome removal. The cDNA was subsequently subjected to qPCR analysis using AceQTM Universal SYBR qPCR Master Mix (Vazyme). *gapA* (a glyceraldehyde-3-phosphate dehydrogenase-encoding gene, *MXAN_2815*) was used as the reference. Here, we measured the linear curve of the concentration vs cycle threshold (Ct) values of each pair of primers and set Ct*_gapA_* = 25 for normalization of each sample.

The quantitative PCR primers used are listed in Table S3.

## SUPPLEMENTAL MATERIAL

Supplemental figures and tables msphere.00363-24-s0001.pdf Figures S1 to S7; Tables S1 to S3.

ASM does not own the copyrights to Supplemental Material that may be linked to, or accessed through, an article. The authors have granted ASM a non-exclusive, world-wide license to publish the Supplemental Material files. Please contact the corresponding author directly for reuse.
