# Supplementary material for: Functional investigation of the two ClpPs and three ClpXs in Myxococcus xanthus DK1622
Source: mSphere. 2024 Aug 27;9(9):e00363-24. doi: 10.1128/msphere.00363-24 (PMC11423568; doi:10.1128/msphere.00363-24)
Supplement: Supplemental figures and tables — Figures S1 to S7; Tables S1 to S3. [file msphere.00363-24-s0001.pdf]

Supplemental Material for

Functional investigation of the two ClpPs and three ClpXs in *Myxococcus xanthus* DK1622

Tianyu Wan[1], Ying Cao[1], Ya-jun Lai[1], Zhuo Pan[1], Yue-zhong Li[1,]*, Li Zhuo[1, 2, 3,]*

*The corresponding authors

Emails: zhuoli1992@sdu.edu.cn, lilab@sdu.edu.cn

This PDF file includes:

Figures S1 to S7

Tables S1 to S3

Supplementary Figures

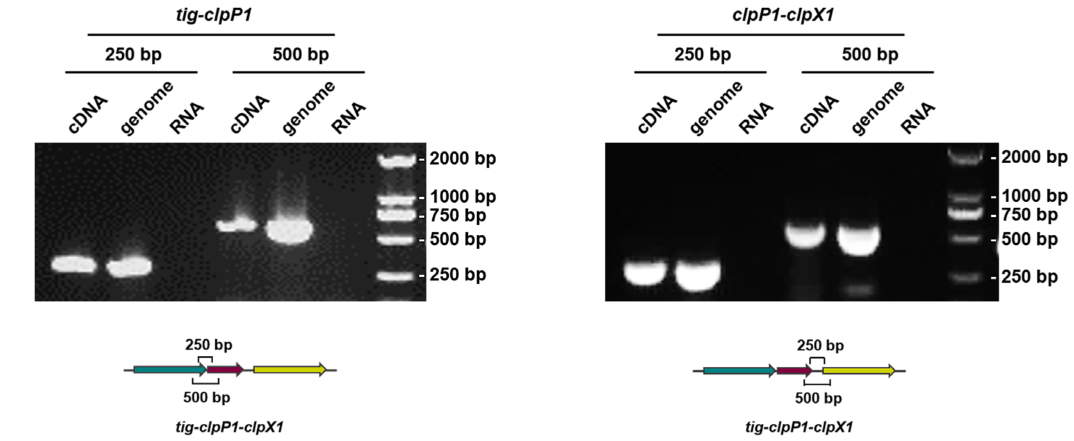

Figure S1. RT-PCR of the intergenic region in *tig-clpP1-clpX1* locus.

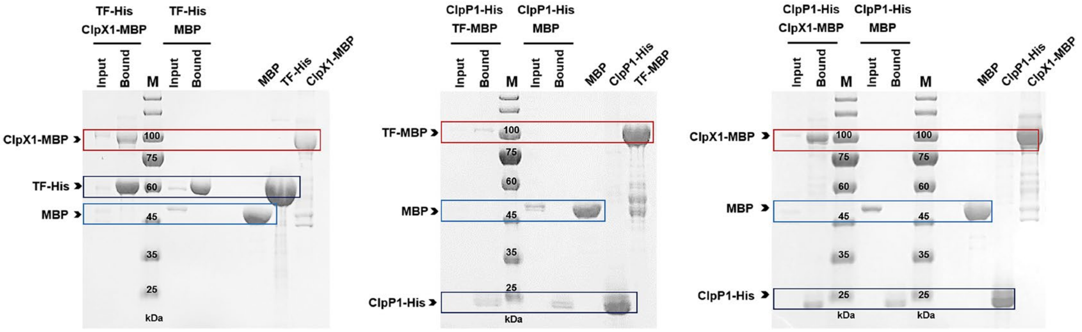

Figure S2. Pull-down of labeled TF, ClpP1 and ClpX1. The system flowed through Ni-NTA, and bound proteins were Ni-NTA binding proteins.

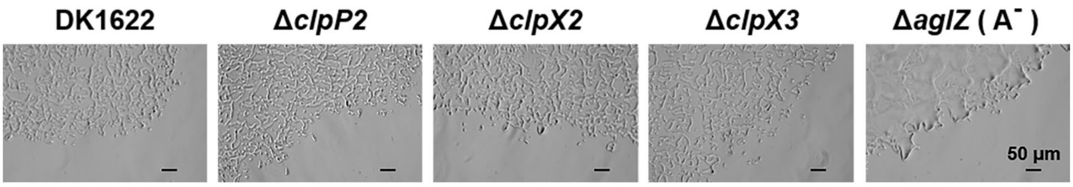

Figure S3. A-motility of *clpP2*, *clpX2*, and *clpX3* deletion mutants. The formation of single colonies distant from the edge of the swarm was observed with a phase contrast microscope. The strain Δ*aglZ* (A^-S^+) was used as a negative control. The bars in black are 50 μm.

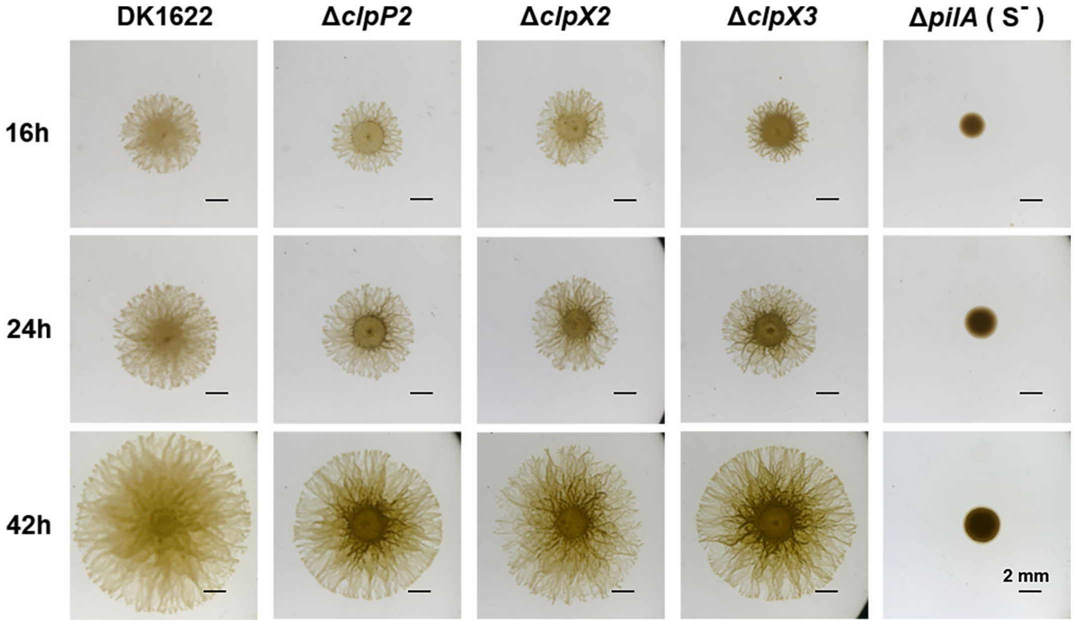

Figure S4. S-motility of *clpP2*, *clpX2*, and *clpX3* deletion mutants. The expansion of the swarm edge on 0.4% agar CTT plates was observed with a stereoscopic microscope at 16 h, 24 h and 42 h. The strain Δ*pilA* (A^+S^-) was used as a negative control. The bars in black are 2 mm.

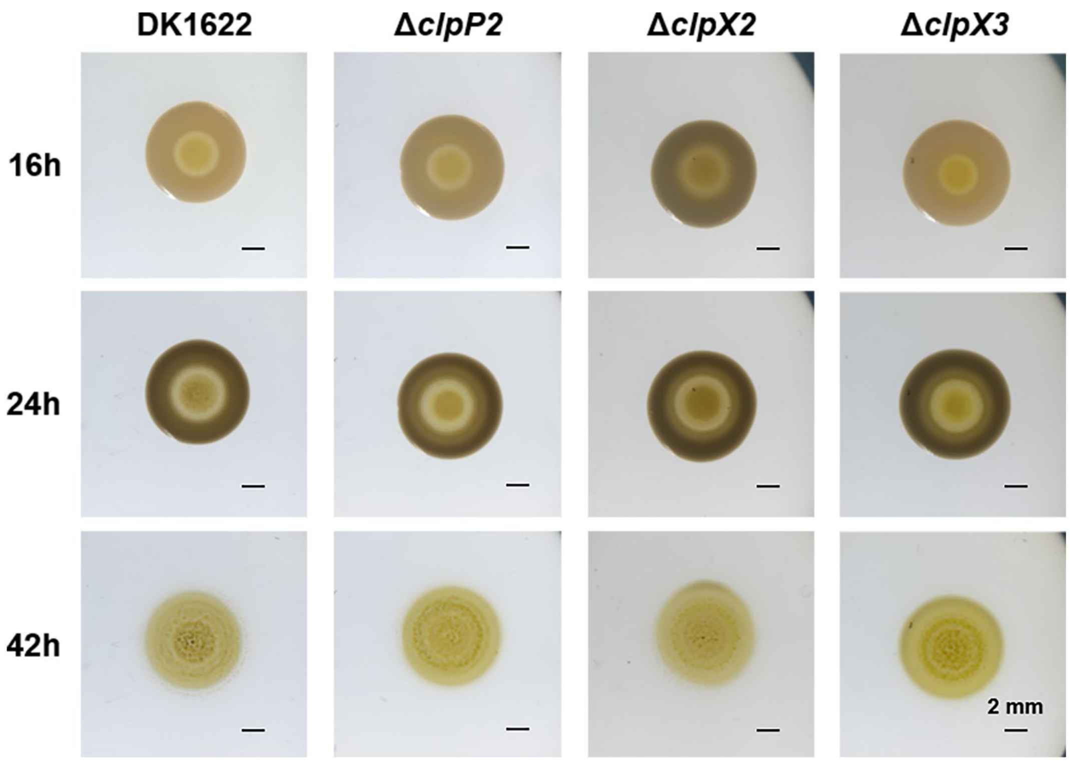

Figure S5. Predation analysis of *clpP2*, *clpX2*, and *clpX3* deletion mutants on *E. coli* prey mats. The predation zones were observed with a stereoscopic microscope at 16 h, 24 h and 42 h. Bars in black are 2 mm.

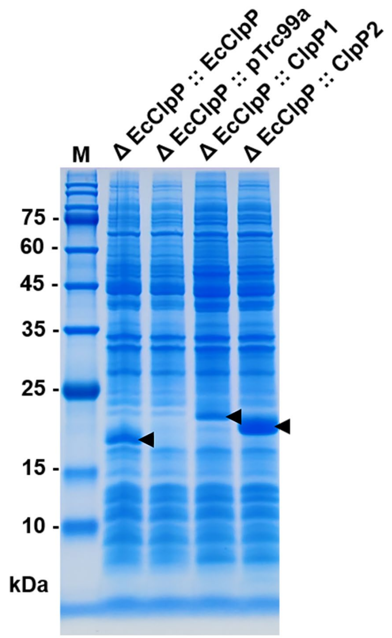

Figure S6. Whole-cell SDS-PAGE of *E. coli* recombinant mutants under the induction of 200 μM IPTG. The induced protein bands are marked by black triangles.

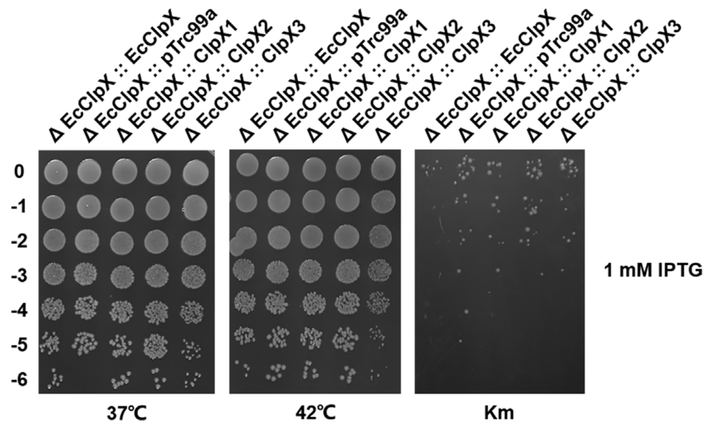

Figure S7. Growth of *E. coli* recombinant mutants heterologously expressing ClpX1, ClpX2 and ClpX3 under different conditions. Then, 1:10 serial dilutions (vertical direction) were spotted on LB plates containing 100 μg/ml Amp with 1 mM IPTG

Supplementary Tables

Table S1. Annotation information of multiple ClpPs and ClpXs in *M. xanthus* DK1622.

<table>
<tr><th></th><th>Name</th><th>Old locus tag</th><th>Locus tag</th><th>Protein product</th><th>Gene length</th><th>Protein length</th><th>Protein name</th><th>Identity to E. coli ClpP/X</th><th colspan="3">Identity</th></tr>
<tr><td rowspan="2">ClpP</td><td>ClpP1</td><td>MXAN_2014</td><td>MXAN_RS09765</td><td>WP_011552098.1</td><td>621</td><td>206</td><td>ATP-dependent Clp protease, proteolytic subunit ClpP</td><td>65.97%</td><td rowspan="2" colspan="3">ClpP1 / ClpP2 79.59%</td></tr>
<tr><td>ClpP2</td><td>MXAN_6438</td><td>MXAN_RS31180</td><td>WP_011556370.1</td><td>612</td><td>203</td><td>ATP-dependent Clp protease, proteolytic subunit ClpP</td><td>65.24%</td></tr>
<tr><td rowspan="3">ClpX</td><td>ClpX1</td><td>MXAN_2015</td><td>MXAN_RS09770</td><td>WP_011552099.1</td><td>1284</td><td>427</td><td>ATP-dependent Clp protease, ATP-binding subunit ClpX</td><td>67.24%</td><td rowspan="3">ClpX2 / ClpX1 64.79%</td><td rowspan="3">ClpX3 / ClpX1 45.35%</td><td rowspan="3">ClpX3 / ClpX2 41.98%</td></tr>
<tr><td>ClpX2</td><td>MXAN_4054</td><td>MXAN_RS19710</td><td>WP_011554061.1</td><td>1278</td><td>425</td><td>ATP-dependent Clp protease, ATP-binding subunit ClpX</td><td>64.82%</td></tr>
<tr><td>ClpX3</td><td>MXAN_2743</td><td>MXAN_RS13295</td><td>WP_026113955.1</td><td>1071</td><td>356</td><td>putative ATP-dependent Clp protease, ATP-binding subunit ClpX</td><td>43.54%</td></tr>
<tr><td colspan="8" rowspan="2"></td><td colspan="4">Identity to E. coli</td></tr>
<tr><td>ClpA</td><td colspan="2">ClpB</td><td>ClpC</td></tr>
<tr><td colspan="2" rowspan="8">ClpA/B/C</td><td>MXAN_6026</td><td>MXAN_RS29235</td><td>ABF92404.1</td><td>2298</td><td>765</td><td>ATP-dependent Clp protease, ATP-binding subunit ClpA</td><td>57.09%</td><td colspan="2">59.16%</td><td>39.69%</td></tr>
<tr><td>MXAN_5092</td><td>MXAN_RS24740</td><td>ABF92187.1</td><td>2625</td><td>874</td><td>ATP-dependent chaperone protein ClpB</td><td>45.14%</td><td colspan="2">57.88%</td><td>48.57%</td></tr>
<tr><td>MXAN_4823</td><td>MXAN_RS23440</td><td>ABF91057.1</td><td>3450</td><td>1149</td><td>ClpB family protein</td><td>30.32%</td><td colspan="2">41.01%</td><td>30.02%</td></tr>
<tr><td>MXAN_4824</td><td>MXAN_RS23445</td><td>ABF88121.1</td><td>3384</td><td>1127</td><td>ClpA/B family protein</td><td>30.54%</td><td colspan="2">37.68%</td><td>32.42%</td></tr>
<tr><td>MXAN_4825</td><td>MXAN_RS23450</td><td>ABF88791.1</td><td>2511</td><td>836</td><td>ClpA/B family protein</td><td>27.80%</td><td colspan="2">36.66%</td><td>39.62%</td></tr>
<tr><td>MXAN_4178</td><td>MXAN_RS20285</td><td>ABF91071.1</td><td>2520</td><td>839</td><td>ClpA/B family protein</td><td>39.41%</td><td colspan="2">48.36%</td><td>42.99%</td></tr>
<tr><td>MXAN_1484</td><td>MXAN_RS07205</td><td>ABF92707.1</td><td>2901</td><td>966</td><td>ClpA/B family protein</td><td>28.37%</td><td colspan="2">36.77%</td><td>39.78%</td></tr>
<tr><td>MXAN_4832</td><td>MXAN_RS23480</td><td>ABF90354.1</td><td>2655</td><td>884</td><td>ATP-dependent Clp protease, ATP-binding subunit ClpC</td><td>38.55%</td><td colspan="2">49.87%</td><td>50.33%</td></tr>
</table>

Table S2. Bacterial strains and plasmids used in this study.

Strain or Plasmid	Genotype or Characteristic(s)	Source or Reference
***E.coli* strains**		
DH5α	*F-φ80 lac ZΔM15 Δ(lacZYA-arg F) U169 endA1 recA1 hsdR17(rk-,mk+) supE44λ- thi -1 gyrA96 relA1 phoA*	Stratagene
BL21	*F_ ompT hsdSB(rB_mB_)dcm gal(DE3)*	Stratagene
MG1655	F- λ- ilvG- rfb-50 rph-1	Stratagene
ΔEcClpP	*E. coli* MG1655 *clpP* deletion strain	This study
ΔEcClpP::pTrc99a	*E. coli* MG1655 Δ*clpP*:: pTrc99a	This study
ΔEcClpP::EcClpP	*E. coli* MG1655 Δ*clpP*:: pTrc99a+*E. coli clpP*	This study
ΔEcClpP::ClpP1	*E. coli* MG1655 Δ*clpP*:: pTrc99a+*M. xanthus MXAN_2014*	This study
ΔEcClpP::ClpP2	*E. coli* MG1655 Δ*clpP*:: pTrc99a+*M. xanthus MXAN_6438*	This study
ΔEcClpX	*E. coli* MG1655 *clpX* deletion strain	This study
ΔEcClpX::pTrc99a	*E. coli* MG1655 Δ*clpX*:: pTrc99a	This study
ΔEcClpX::EcClpX	*E. coli* MG1655 Δ*clpX*:: pTrc99a+*E. coli clpX*	This study
ΔEcClpX::ClpX1	*E. coli* MG1655 Δ*clpX*:: pTrc99a+*M. xanthus MXAN_2015*	This study
ΔEcClpX::ClpX2	*E. coli* MG1655 Δ*clpX*:: pTrc99a+*M. xanthus MXAN_4054*	This study
ΔEcClpX::ClpX3	*E. coli* MG1655 Δ*clpX*:: pTrc99a+*M. xanthus MXAN_2743*	This study
***M.xanthus* strains**		
DK1622	Wild-type strain	D.Kaiser University of Standford
DK1622 *att*::pSWU30	*M. xanthus* DK1622:: pSWU30	This study
DK1622 *att*::P_{pilA}+*clpP1*	*M. xanthus* DK1622:: pSWU30+P_{pilA}+*MXAN_2014*	This study
DK1622 *att*::P_{pilA}+*clpP2*	*M. xanthus* DK1622:: pSWU30+P_{pilA}+*MXAN_6438*	This study
DK1622 *att*::P_{pilA}+*clpX1*	*M. xanthus* DK1622:: pSWU30+P_{pilA}+*MXAN_2015*	This study

DK1622 *att*::P_{pilA}+*clpX2*	*M. xanthus* DK1622:: pSWU30+P_{pilA}+*MXAN_4054*	This study
DK1622 *att*::P_{pilA}+*clpX3*	*M. xanthus* DK1622:: pSWU30+P_{pilA}+*MXAN_2743*	This study
DK1622 *att*::P_{pilA}+*clpP1* Δ*clpP1*	*M. xanthus* DK1622 *att*::P_{pilA}+*MXAN_2014* then knock-out *MXAN_2014*	This study
DK1622 *att*::*PpilA*+*clpX1* Δ*clpX1*	*M. xanthus* DK1622 *att*::P_{pilA}+*MXAN_2015* then knock-out *MXAN_2015*	This study
Δ*clpP2*	DK1622 knock-out *MXAN_6438*	This study
Δ*clpX2*	DK1622 knock-out *MXAN_4054*	This study
Δ*clpX3*	DK1622 knock-out *MXAN_2743*	This study
Δ*pilA*	DK1622 Δ*MXAN_5783* (DK10410)	D.Kaiser University of Standford
Δ*aglZ*	DK1622 Δ*MXAN_2991* (MxH2265)	P. Hartzell University of Idaho
Δ *clpX3*::*clpX3*	DK1622 knock-out *MXAN_2743* then compensate *MXAN_2743* with P_{pilA} at *attB* site	This study
Δ *clpX3*::*clpX2*	DK1622 knock-out *MXAN_2743* then compensate *MXAN_4054* with P_{pilA} at *attB* site	This study
Δ *clpX3*::*clpX2*Δ*N*	DK1622 knock-out *MXAN_2743* then compensate N-terminal deleted *MXAN_4054* with P_{pilA} at *attB* site	This study
Δ *clpX2*::*clpX2*	DK1622 knock-out *MXAN_4054* then compensate *MXAN_2743* added N-terminal of *MXAN_4054* with P_{pilA} at *attB* site	This study
Δ *clpX2*::*clpX2*Δ*N*	DK1622 knock-out *MXAN_4054* then compensate N-terminal deleted *MXAN_4054* with P_{pilA} at *attB* site	This study
Δ *clpX2*::*clpX3*	DK1622 knock-out *MXAN_4054* then compensate *MXAN_2743* with P_{pilA} at *attB* site	This study
Δ *clpX2*::*clpX3*+N_{clpX2}	DK1622 knock-out *MXAN_4054* then compensate *MXAN_2743* with P_{pilA} at *attB* site	This study
**Plasmids**		
pTrc99a	IPTG induced expression vector in *E. coli* (AmpR)	Laboratory collection
pTrc-EcClpP	IPTG induced expression vector of *E. coli clpP* (AmpR)	This study
pTrc-MXAN_2014	IPTG induced expression vector of *M. xanthus MXAN_2014* (AmpR)	This study
pTrc-MXAN_6438	IPTG induced expression vector of *M. xanthus MXAN_6438* (AmpR)	This study
pTrc-EcClpX	IPTG induced expression vector of *E. coli clpP* (AmpR)	This study
pTrc-MXAN_2015	IPTG induced expression vector of *M. xanthus MXAN_2015* (AmpR)	This study
pTrc-MXAN_4054	IPTG induced expression vector of *M. xanthus MXAN_4054* (AmpR)	This study
pTrc-MXAN_2743	IPTG induced expression vector of *M. xanthus MXAN_2743* (AmpR)	This study

pET28a	Expression vector (KmR)	Laboratory collection
pET28a-MXAN_2014	Expression vector of MXAN_2014-His (KmR)	This study
pET28a-MXAN_6438	Expression vector of MXAN_6438-His (KmR)	This study
pET28a-MXAN_2015	Expression vector of MXAN_2015-His (KmR)	This study
pET28a-MXAN_4054	Expression vector of MXAN_4054-His (KmR)	This study
pET28a-MXAN_2743	Expression vector of MXAN_2743-His (KmR)	This study
pMal-c5x	Expression vector with with the solubility-enhancing tag MBP (AmpR)	Laboratory collection
pMal-MXAN_2014	Expression vector of MXAN_2014-MBP (AmpR)	This study
pMal-MXAN_6438	Expression vector of MXAN_6438-MBP (AmpR)	This study
pMal-MXAN_2015	Expression vector of MXAN_2015-MBP (AmpR)	This study
pMal-MXAN_4054	Expression vector of MXAN_4054-MBP (AmpR)	This study
pMal-MXAN_2743	Expression vector of MXAN_2743-MBP (AmpR)	This study
pSWU30	*attB* site-specific recombination vector of *M. xanthus* (TetR)	Laboratory collection
pSWU30-PpilA+MXAN_2014	Overexpression plasmid of *MXAN_2014* by PpilA promoter (TetR)	This study
pSWU30-PpilA+MXAN_6438	Overexpression plasmid of *MXAN_6438* by PpilA promoter (TetR)	This study
pSWU30-PpilA+MXAN_2015	Overexpression plasmid of *MXAN_2015* by PpilA promoter (TetR)	This study
pSWU30-PpilA+MXAN_4054	Overexpression plasmid of *MXAN_4054* by PpilA promoter (TetR)	This study
pSWU30-PpilA+MXAN_2743	Overexpression plasmid of *MXAN_2743* by PpilA promoter (TetR)	This study
pSWU19	*attB* site-specific recombination vector of *M. xanthus* (KmR)	Laboratory collection
pSWU19-PpilA+MXAN_2743	Expression plasmid of *MXAN_2743* by PpilA promoter in *M. xanthus* (KmR)	This study
pSWU19-PpilA+MXAN_4054	Expression plasmid of *MXAN_4054* by PpilA promoter in *M. xanthus* (KmR)	This study
pSWU19-PpilA+MXAN_4054ΔN	Expression plasmid of N-terminal deleted *MXAN_4054* by PpilA promoter in *M. xanthus* (KmR)	This study
pSWU19-PpilA+MXAN_2743+N_{MXAN_4054}	Expression plasmid of *MXAN_2743* with N-terminal of *MXAN_4054* by PpilA promoter in *M. xanthus* (KmR)	This study
pBJ113	Gene replacement vector with KG cassette (KmR)	Laboratory collection
pBJ-MXAN_2014 del	Upstream and downstream homologous arms of DK1622 *MXAN_2014* and inserted into MCS of pBJ113 (KmR)	This study

pBJ-MXAN_6438 del	Upstream and downstream homologous arms of DK1622 *MXAN_6438* and inserted into MCS of pBJ113 (KmR)	This study
pBJ-MXAN_2015 del	Upstream and downstream homologous arms of DK1622 *MXAN_2015* and inserted into MCS of pBJ113 (KmR)	This study
pBJ-MXAN_4054 del	Upstream and downstream homologous arms of DK1622 *MXAN_4054* and inserted into MCS of pBJ113 (KmR)	This study
pBJ-MXAN_2743 del	Upstream and downstream homologous arms of DK1622 *MXAN_2743* and inserted into MCS of pBJ113 (KmR)	This study

Table S3. List of primers used in this study.

Primer	Sequence (5-3)	Use
pBJ113-F	GGGGATCCTCTAGAGTCGACCT	PCR amplification of pBJ13 fragment for construction of gene deletion vectors
pBJ113-R	GGGTACCGAGCTCGAATTCA	
MXAN_2014-UF	tgaattcgagctcggtacccGACGAGCAGGTGAACGAGCA	PCR amplification of upstream homologous arm for deletion of *MXAN_2014*
MXAN_2014-UR	caatccgcGTCGACCTCGGTGAGGGAA	
MXAN_2014-DF	accgaggtcgacGCGGATTGCCGAGGCTTC	PCR amplification of downstream homologous arm for deletion of *MXAN_2014*
MXAN_2014-DR	gtcgactctagaggatccccCCGCCGCAGATGAAGAGG	
MXAN_6438-UF	tgaattcgagctcggtacccGTCGGCGGCTTCGTGGCG	PCR amplification of upstream homologous arm for deletion of *MXAN_6438*
MXAN_6438-UR	gccGACGCCCCTACCCTAATCGC	
MXAN_6438-DF	attagggtaggggcgtcGGCCCTTCCCGCCCCGGA	PCR amplification of downstream homologous arm for deletion of *MXAN_6438*
MXAN_6438-DR	gtcgactctagaggatccccCTCGCAGTGGCGGAGCGG	
MXAN_2015-UF	tgaattcgagctcggtacccAGCAGCTCGCCAACGAGG	PCR amplification of upstream homologous arm for deletion of *MXAN_2015*
MXAN_2015-UR	cctggcgTTCGTCCCTCGCTGCGCC	
MXAN_2015-DF	agcgagggacgaaCGCCAGGGCCCGTAGGGG	PCR amplification of downstream homologous arm for deletion of *MXAN_2015*
MXAN_2015-DR	gtcgactctagaggatccccACGGTGTCCTTCGACGGC	
MXAN_4054-UF	tgaattcgagctcggtacccCTGCTGTCGGAGATGCAGAAG	PCR amplification of upstream homologous arm for deletion of *MXAN_4054*
MXAN_4054-UR	agaagggagaccgccgTGCCGCCTCCGAGTTCGC	
MXAN_4054-DF	ggcaCGGCGGTCTCCCTTCTCC	PCR amplification of downstream homologous arm for deletion of *MXAN_4054*
MXAN_4054-DR	gtcgactctagaggatccccCCATCTCCCTGGAGAACGC	
MXAN_2743-UF	tgaattcgagctcggtacccATCAGCTCGGAGGAGGTCAGG	PCR amplification of upstream homologous arm for deletion of *MXAN_2743*
MXAN_2743-UR	tgatGTACCCTCCCCTTGTTTCCACG	
MXAN_2743-DF	aacaaggggagggtacATCAGGGCTCGCGCTCGG	PCR amplification of downstream homologous arm for deletion of *MXAN_2743*
MXAN_2743-DR	gtcgactctagaggatccccAAGAGCAGATCGGCACCCA	

pSWU30-U	AGAGGATCCCCGGGTACCG	PCR amplification of pSWU30 fragment for construction of gene expression vectors
pSWU30-D	AGAGTCGACCTGCAGGCATG	
pSWU30-PpilA-U	GTGGAGCGGGCCGCGACC	PCR amplification of PpilA fragment for construction of gene expression vectors
pSWU30-PpilA-D	GGGGGTCCTCAGAGAAGGTTG	
pSWU30-A-2014-U	aggacccccATGCCCTTCATGCCCGTT	PCR amplification of MXAN_2014 for ligation with pSWU30-PpilA
pSWU30-A-2014-D	tcggtacccggggatcctctCTACTTCTTCTCTTCCTTACCCAGTCC	
pSWU30-A-6438-U	aaccttctctgaggacccccATGAACGTCCCCTTCGTCATC	PCR amplification of MXAN_6438 for ligation with pSWU30-PpilA
pSWU30-A-6438-D	ggtacccggggatcctctagCTACTTCGCCGGAGCGGG	
pSWU30-A-2015-U	ttctctgaggacccccATGGCGGGCAAGAACGTG	PCR amplification of MXAN_2015 for ligation with pSWU30-PpilA
pSWU30-A-2015-D	tcggtacccggggatcctctTCAGGCGGATTCCTTCTTCG	
pSWU30-A-4054-U	aaccttctctgaggacccccGTGAAGAAGGAGCACCACGTCA	PCR amplification of MXAN_4054 for ligation with pSWU30-PpilA
pSWU30-A-4054-D	ggtacccggggatcctctagCTAGGCCGTCTTCTTCTCCTTCT	
pSWU30-A-2743-U	aaccttctctgaggacccccATGGAGTCGTCCGCACGC	PCR amplification of MXAN_2743 for ligation with pSWU30-PpilA
pSWU30-A-2743-D	ggtacccggggatcctctagTCACACGTCCAACTGCGTCG	
pSWU19-U	AGAGGATCCCCGGGTACCG	PCR amplification of pSWU19 fragment for construction of gene expression vectors
pSWU19-D	AGAGTCGACCTGCAGGCATG	
pSWU19-PpilA-U	GTGGAGCGGGCCGCGACC	PCR amplification of PpilA fragment for construction of gene expression vectors
pSWU19-PpilA-D	GGGGGTCCTCAGAGAAGGTTG	
pSWU19-A-4054-U	aaccttctctgaggacccccGTGAAGAAGGAGCACCACGTCA	PCR amplification of MXAN_4054 for ligation with pSWU19-PpilA
pSWU19-A-4054-D	ggtacccggggatcctctagCTAGGCCGTCTTCTTCTCCTTCT	
pSWU19-A-2743-U	aaccttctctgaggacccccATGGAGTCGTCCGCACGC	PCR amplification of MXAN_2743 for ligation with pSWU19-PpilA
pSWU19-A-2743-D	ggtacccggggatcctctagTCACACGTCCAACTGCGTCG	
pSWU19-A-4054ΔN-U	aaccttctctgaggacccccGTGAAGAAGGAGCACCACGTCA	PCR amplification of MXAN_4054ΔN for ligation with pSWU19-PpilA
pSWU19-A-4054ΔN-D	atttcccgcggggtcagcagGCTGACCTGGGGCTTGCC	
pSWU19-A-2743+N-U	CTGCTGACCCCGCGGGAA	PCR amplification of MXAN_2743+N_{MXAN_4054} for ligation with pSWU19-PpilA

pSWU19-A-2743+N-D	GGGGGTCCTCAGAGAAGGTTG	
pTrc99a-U	GAGAAGATTTTCAGCCTGATACAGATT	PCR amplification of pTrc99a fragment for construction of gene expression vectors
pTrc99a-D	GGTCTGTTTCCTGTGTGAAATTGT	
eClpP-pTrc-U	tttcacacaggaaacagaccATGTCATACAGCGGCGAACG	PCR amplification of *E.coli clpP* for ligation with pTrc99a
eClpP-pTrc-D	atcaggctgaaaatcttctcTCAATTACGATGGGTCAGAATC	
2014-pTrc-U	tttcacacaggaaacagaccATGAAGGTCCAGGTCGAGGAG	PCR amplification of *MXAN_2014* for ligation with pTrc99a
2014-pTrc-D	atcaggctgaaaatcttctcTTACGAATACTTCGCCCGGC	
6438-pTrc-U	tttcacacaggaaacagaccATGAACGTCCCCTTCGTCATC	PCR amplification of *MXAN_6438* for ligation with pTrc99a
6438-pTrc-D	atcaggctgaaaatcttctcCTACTTCGCCGGAGCGGG	
eClpX-pTrc-U	ttcacacaggaaacagaccATGACAGATAAACGCAAAGATGG	PCR amplification of *E.coli clpX* for ligation with pTrc99a
eClpX-pTrc-D	atcaggctgaaaatcttctcTTATTCACCAGATGCCTGTTGC	
2015-pTrc-U	tttcacacaggaaacagaccATGGCGGGCAAGAACGTG	PCR amplification of *MXAN_2015* for ligation with pTrc99a
2015-pTrc-D	atcaggctgaaaatcttctcTCAGGCGGATTCCTTCTTCG	
4054-pTrc-U	tttcacacaggaaacagaccGTGAAGAAGGAGCACCACGTCA	PCR amplification of *MXAN_4054* for ligation with pTrc99a
4054-pTrc-D	atcaggctgaaaatcttctcCTAGGCCGTCTTCTTCTCCTTC	
2743-pTrc-U	tttcacacaggaaacagaccATGGAGTCGTCCGCACGC	PCR amplification of *MXAN_2743* for ligation with pTrc99a
2743-pTrc-D	atcaggctgaaaatcttctcTCACACGTCCAACTGCGTCG	
pET28a-U	GAATTCGAGCTCCGTCGACA	PCR amplification of pET28a fragment for construction of gene expression vectors
pET28a-D	GGATCCGCGACCCATTTG	
pET28a-2014-U	agcaaatgggtcgcggatccATGCCCTTCATGCCCGTT	PCR amplification of MXAN_2014 for ligation with pET28a
pET28a-2014-D	tgtcgacggagctcgaattcCTACTTCTTCTCTTCCTTACCCAGTCC	
pET28a-6438-U	agcaaatgggtcgcggatccATGAACGTCCCCTTCGTCATC	PCR amplification of MXAN_6438 for ligation with pET28a
pET28a-6438-D	tgtcgacggagctcgaattcCTACTTCGCCGGAGCGGG	
pET28a-2015-U	agcaaatgggtcgcggatccATGGCGGGCAAGAACGTG	PCR amplification of MXAN_2015 for ligation with pET28a
pET28a-2015-D	tgtcgacggagctcgaattcTCAGGCGGATTCCTTCTTCG	

pET28a-4054-U	agcaaatgggtcgcggatccGTGAAGAAGGAGCACCACGTCA	PCR amplification of MXAN_4054 for ligation with pET28a
pET28a-4054-D	tgtcgacggagctcgaattcCTAGGCCGTCTTCTTCTCCTTCT	
pET28a-2743-U	agcaaatgggtcgcggatccATGGAGTCGTCCGCACGC	PCR amplification of MXAN_2743 for ligation with pET28a
pET28a-2743-D	tgtcgacggagctcgaattcTCACACGTCCAACTGCGTCG	
pMal-U	CGCGGATCCGAATTCGAGCT	PCR amplification of pMal-c5x fragment for construction of gene expression vectors
pMal-D	GGGCCCCTGGAACAGAACTTC	
pMal-2014-U	aagttctgttccaggggcccATGCCCTTCATGCCCGTT	PCR amplification of MXAN_2014 for ligation with pMal-c5x
pMal-2014-D	agctcgaattcggatccgcgCTACTTCTTCTCTTCCTTACCCAGTCC	
pMal-6438-U	aagttctgttccaggggcccATGAACGTCCCCTTCGTCATC	PCR amplification of MXAN_6438 for ligation with pMal-c5x
pMal-6438-D	agctcgaattcggatccgcgCTACTTCGCCGGAGCGGG	
pMal-2015-U	aagttctgttccaggggcccATGGCGGGCAAGAACGTGGA	PCR amplification of MXAN_2015 for ligation with pMal-c5x
pMal-2015-D	agctcgaattcggatccgcgTCAGGCGGATTCCTTCTTCG	
pMal-4054-U	aagttctgttccaggggcccGTGAAGAAGGAGCACCACGT	PCR amplification of MXAN_4054 for ligation with pMal-c5x
pMal-4054-D	agctcgaattcggatccgcgCTAGGCCGTCTTCTTCTCCT	
pMal-2743-U	aagttctgttccaggggcccATGGAGTCGTCCGCACGCAG	PCR amplification of MXAN_2743 for ligation with pMal-c5x
pMal-2743-D	agctcgaattcggatccgcgTCACACGTCCAACTGCGTCG	
2014-q-U	GATGAAGGCGAAGCTCAACGAG	Transcription detection of MXAN_2014
2014-q-D	TTCTCTTCCTTACCCAGTCCCAC	
6438-q-U	CCTGCGCAGCTACATCAACGGC	Transcription detection of MXAN_6438
6438-q-D	GCGCTGCTTCTCCACCAC	
2015-q-U	ACAAGATTGCACGCAAGTCG	Transcription detection of MXAN_2015
2015-q-D	CGCTCAATCACCTGGTCCAG	
4054-q-U	AAGTGGAGCTGAGCAAGAGCA	Transcription detection of MXAN_4054
4054-q-D	TCGGCGTTGTGGAGGAGGT	
2743-q-U	TCGCTCATCAAGAAATCCAACA	Transcription detection of MXAN_2743

2743-q-D	TCCTCCACGGAGTGGTTCGC	
gapA-q-U	GCCCTGGAAGAGCCTGAACG	Reference gene for transcription detection
gapA-q-D	TCGAGATGACGTGGTGCTTGG	
